# Reliability Modeling Method for Lithium-ion Battery Packs Considering the Dependency of Cell Degradations Based on a Regression Model and Copulas

**DOI:** 10.3390/ma12071054

**Published:** 2019-03-30

**Authors:** Lizhi Wang, Yusheng Sun, Xiaohong Wang, Zhuo Wang, Xuejiao Zhao

**Affiliations:** 1 Institute of Unmanned System, Beihang University, Beijing 100191, China; wanglizhi@buaa.edu.cn; 2 Key Laboratory of Advanced Technology of Intelligent Unmanned Flight System of Ministry of Industry and Information Technology, Beijing 100191, China; 3 School of Reliability and Systems Engineering, Beihang University, Beijing 100191, China; sunyusheng@buaa.edu.cn (Y.S.); 13121698585@163.com (Z.W.); zhaoxuejiao@buaa.edu.cn (X.Z.)

**Keywords:** lithium-ion battery pack, dependency, reliability models considering dependency, copula function

## Abstract

Lithium-ion batteries are widely used as basic power supplies and storage units for large-scale electric drive products such as electric vehicles. Their reliability is directly related to the life and safe operation of the electric drive products. In fact, the cells have a dependent relationship with the degradation process and they affect the degradation rate of the entire battery pack, thereby affecting its reliability. At present, most research focuses on the reliability of battery packs and assumes that their cells are independent of each other, which may cause the reliability of the evaluation to deviate greatly from the actual level. In order to accurately assess the reliability of lithium-ion batteries, it is necessary to build a reliability model considering the dependency among cells for the overall degradation of lithium-ion battery packs. Therefore, in this study, based on a lithium-ion battery degradation test, the Wiener process is used to analyze the reliability of four basic configurations of lithium-ion battery packs. According to the degradation data of the battery packs, the Copula function is used to quantitatively describe the dependent relationship in the degradation process of a single battery, and the quantitative dependent relationship is combined with the reliability model to form a new reliability model. Finally, taking the battery system of Tesla S as an example, a feasible optimization method for battery pack design is provided based on the model constructed in this work.

## 1. Introduction

In recent years, lightweight energy storage devices such as lithium-ion batteries and supercapacitors have been widely utilized in electric vehicles, aerospace applications, submarines, and other power fields [1]. As an important clean energy source, lithium-ion batteries have advantages of high energy density, high capacity, low self-discharge rate, good safety, long life, and no pollution. Usually, hundreds of cells are connected, both in series and in parallel, to form battery packs in order to meet the voltage and energy requirements of electric drive products. The reliability of the battery packs is directly related to the overall performance of the electric drive products. For this reason, accurate knowledge of the reliability of the battery packs is vital in order to ensure the long life of the electric drive product. Previous studies have found that the dependency among cells during battery charging and discharging, as well as the degradation of each cell, will affect the degradation process of the associated single battery. Because of the dependency among cells, the degradation amount and the degradation rate of a battery pack are neither equal to those of the cells nor to the sum of those of cells [2]. Accordingly, the degradation process of a battery pack depends not only on the performance of a single battery, but also on the dependency among the individual cells. Therefore, it is necessary to reevaluate the failure law of lithium-ion battery packs under the influence of the dependency among cells and to conduct a reliability analysis of the battery packs.

Currently, several studies on the reliability of lithium-ion batteries are based on the reliability analysis method, which assumes that the cells in a battery pack are independent of each other [3,4]. Li et al. believe that this interrelationship among cells could affect the performance and lifetime of a battery pack [5]. Other scholars have discovered this inconsistency among cells in a lithium-ion battery pack and have studied its effects. Zheng et al. studied the influence of the inconsistency of the charging status on the safety and durability of lithium-ion batteries. The second-order RC model was chosen to represent the overall performance of the lithium-ion batteries and the inconsistency of the charging status of the lithium-ion batteries was estimated [6]. Wang et al. established a capacity degradation model for series battery packs in the case of inconsistent cell capacity [7]. Zhang et al. considered the influence of battery pack inconsistency caused by different operating temperatures and proposed a new method for estimating the residual discharge energy of battery packs using the recursive least squares unscented Kalman filter [8]. In order to improve the inconsistency of the series lithium-ion battery, Ma et al. proposed the use of fuzzy logic control (FLC) to reduce battery pack energy consumption and equalization time based on a non-dissipative equalization scheme [9]. Wang et al. carried out degradation tests on battery packs connected in series, in parallel, in series-parallel, and in parallel-series, and established a capacity degradation model considering the dependency of battery packs [2]. In summary, most of the existing work on the reliability of lithium-ion batteries does not consider the influence of dependency among cells, which leads to low accuracy of reliability estimation. Published research that considers the inconsistencies of battery packs are mostly related to specific battery pack configurations [6,7,8,9]. These studies have built capacity degradation models, but they lack reliability models between cells and the entire battery pack that consider dependency [2,7]. Hence, this work focuses on a reliability model that considers the dependency among cells for various battery pack configurations.

In view of the aforementioned reliability modeling problem, Sarra et al. regarded windmills and gearboxes as multi-component systems. The behavior of one component affected the behavior of the other and the performance of the system [10], which is essentially similar to the dependency phenomenon among cells of the battery pack. Therefore, a lithium-ion battery pack can also be viewed as a multi-component system, in which each cell is regarded as a component. The dependency analysis method applied in a multi-component system can therefore be applied to the dependency modeling of a lithium-ion battery pack. By investigating the methods of dependency quantitative analysis, we found that the Copula function is a widely used statistical tool for describing the dependency and reliability of multi-component systems, and this function can be used in the research of dependency measurements [11]. Yi et al. first applied the Copula function to a discussion of the dependent relationship in reliability theory. The Copula function was used to clarify the dependency of each component of the system and the influence of dependency on the system [12]. Liu et al. presented a reliability evaluation based on the Copula function for an s-dependent degradation process system, and they used the inverse Gaussian process with a time-scale transformation to model the edge degradation process [13]. Hao et al. utilized the Copula function to represent the dependency of the two characteristic curves in a system [14]. The Copula function has also been successfully applied in correlation analysis of financial analysis and risk management [15,16,17], but it has rarely been applied to the study of dependency among cells of lithium-ion battery packs.

In this work, a reliability model of a lithium-ion battery pack is constructed, considering the dependency among cells. Based on the capacity degradation data of the battery packs, the dependency effect on the reliability of the battery packs is verified by the calculation results of the reliability block diagram (RBD) model and the actual test data. Additionally, the Copula function is used to quantify the dependency among cells. Furthermore, a new model is formed by combining the quantified dependency with the RBD model, considering a structural relationship. Finally, the fitting effect of the proposed model is verified with linear regression analysis.

The remaining paper is organized as follows. The reliability analysis of the lithium-ion battery pack with consideration of the influence of dependency among cells is given in Section 2. In Section 3, the dependency among cells is calculated based on the Copula function, and the reliability model that considers dependency among cells of a lithium-ion battery is established. Section 4 shows the application of the reliability model in the group design of a lithium-ion battery pack. Conclusions are presented in Section 5.

## 2. The Dependency Phenomenon and its Influence on a Lithium-Ion Battery Pack

### 2.1. Degradation Test

**Figure 1:**
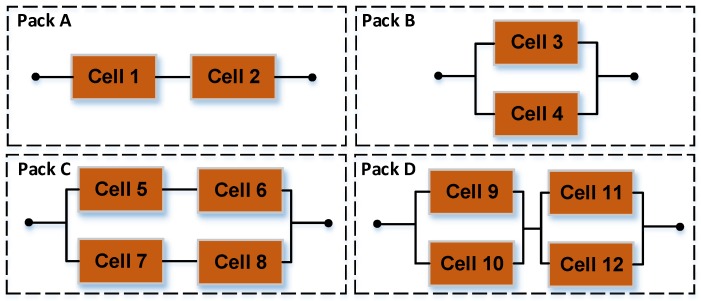
Configurations of the four battery packs.

**Figure 2:**
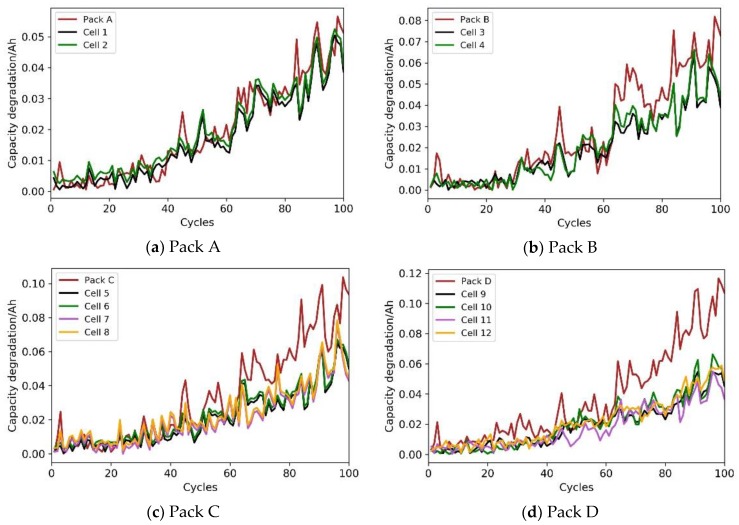
Capacity degradation increment curves of the four battery packs: (**a**) Pack A, (**b**) Pack B, (**c**) Pack C, (**d**) Pack D.

To study the dependency among cells, it is necessary to obtain the actual degradation data of cells and battery packs. In this work, the degradation test and the schematic diagram of battery packs were designed according to the degradation test platform in reference [2]. Degradation tests were carried out on the lithium-ion battery packs in the four battery packs of Pack A (series configuration), Pack B (parallel configuration), Pack C (series and parallel configuration), Pack D (parallel and series configuration), as shown in Figure 1. Through analysis of the current and voltage data collected during the charging and discharging process, it was found that dependency results from the phenomena of overcharge, overcurrent, overvoltage, and circulating current for a single battery with an increasing number of cycles. The detailed mechanism discussion about the dependency analysis in the charge-discharge cycle and dependency analysis in the degradation process were shown in Section 3 of reference [2]. In this work, capacity was taken as the characteristic quantity of the battery’s performance, and the capacity degradation increment curves of the four battery packs and of all cells after 100 cycles were obtained, as shown in Figure 2.

Figure 2 shows that the dependency degree among cells was positively correlated with the overall degradation rate of the battery pack, and that the more complex the structure of the battery pack, the greater the degree of dependency. The battery dependency accelerated the degradation of the whole battery pack, and the degradation process of the battery pack was directly related to its reliability. Therefore, our work re-studied the failure law and analyzed the reliability of a lithium-ion battery.

### 2.2. Reliability Analysis of a Lithium Ion Battery Pack

According to the data obtained from the battery pack degradation test, the performance degradation of the lithium-ion battery pack and the single battery at a certain time was random; therefore, the Wiener process could be used to describe the trend of battery capacity degradation. Some studies have used the Wiener process to model and evaluate the degradation process of lithium-ion batteries. Tang used the non-linear Wiener process to predict the remaining useful life (RUL) of lithium-ion batteries by combining the classical Maximum Likelihood Estimate (MLE) and the Bayesian updating algorithm [18]. The Wiener process is a continuous-time stochastic process that can typically simulate the stochastic degradation process of lithium-ion batteries. The expression for the Wiener process is:

(1) $$Y=\sigma B(t)+\lambda t+y_{0}$$

where Y denotes the capacity degradation of a battery, B(t) denotes the standard Brownian motion, B(t)~N(0,t), σ denotes the diffusion coefficient, σ > 0, *λ* denotes the drift coefficient, and *y*_0_ denotes the capacity degradation value of the battery at initial time *t*_0_, which is generally assumed to be 0. If *w* is the failure threshold of the lithium-ion battery, the battery will cease to be effective when *Y* − *w* < 0. Generally, 80% of the rated capacity of a battery is taken as its failure threshold value. The failure thresholds of the four packs and the cell are given in Table 1. The time (t) at which Y passes through *w* for the first time is subject to an inverse Gaussian distribution, so the distribution of the reliability probability is defined as:

(2) $$R=\Phi \left(\frac{w-\lambda t}{\sigma \sqrt{t}}\right)-\exp\left\{\frac{2\lambda \cdot w}{\sigma^{2}}\right\}\Phi \left(-\frac{w+\lambda t}{\sigma \sqrt{t}}\right)$$

The Wiener process can be used to calculate the reliability of battery packs and cells under actual conditions. However, in the design stage of battery packs, there is usually a lack of degradation data for the whole pack. When analyzing the reliability of the whole pack, the reliability model is typically constructed with a reliability block diagram (RBD). For example, Sun analyzed the reliability of the battery packs in series and in parallel with reliability theory, and it was concluded that the reliability of the battery pack increased with an increasing number of parallel cells [19]. Liu et al. used a reliability assessment model to estimate the Mean Time Between Failures (MTBF), and it was discovered that the reliability of a parallel-series battery pack was better than that of the series-parallel battery pack [20]. The reliability models of series and parallel structures are given in [21]:

(3) $$\mathrm{Series}:\;R=\prod_{i=1}^{n}R_{i}\left(t\right)$$

(4) $$\mathrm{Parallel}:\;R=1-\prod_{i=1}^{n}\left[1-R_{i}\left(t\right)\right]$$

**Figure 3:**
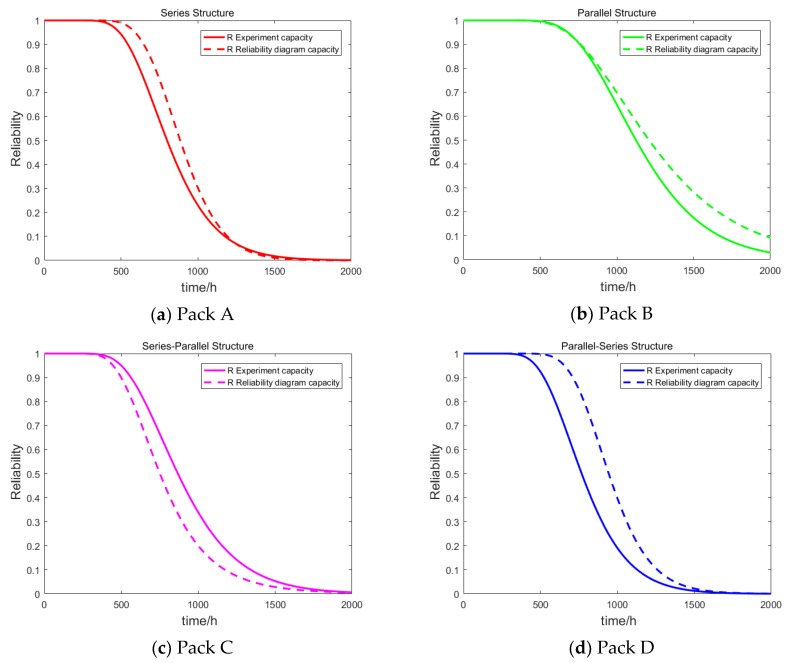
Comparison of reliability of the RBD model with the real reliability of the four battery packs: (**a**) Pack A, (**b**) Pack B, (**c**) Pack C, (**d**) Pack D.

The reliability of complex grouped structures can be calculated by a combination of series and parallel reliability Equations. Using the reliability function in Table 2, the reliability of the four battery packs (Pack A, Pack B, Pack C, and Pack D) without consideration of the dependency among cells can be obtained. The RBD model assumes that the individual cells are independent of each other, regardless of the internal dependency among cells or the overall reliability of the battery pack. Based on the obtained test data of the cell, the reliabilities of Pack A, Pack B, Pack C, and Pack D battery packs were estimated with the RBD model. At the same time, the real reliability of the four battery packs was obtained according to the degradation data of the whole battery packs monitored in practice. The results of the reliability block diagram model and the actual reliability are shown in Figure 3.

In Figure 3, the reliabilities of the four lithium-ion battery packs obtained with the RBD model are compared with those obtained by the actual whole battery pack test data. The actual reliability of the four battery packs was different than the reliability obtained with the RBD method, indicating that the dependency between the capacity degradation processes of the individual cells had a significant impact on the reliability of the battery pack. The difference between the “R Experiment capacity” curve and the “R Reliability diagram capacity” curve represents the influence of the dependency among cells on the reliability of the battery packs. Table 3 compares the RMSE values of the reliability estimated by the test data with values estimated by the RBD model. The RMSE values of the series-parallel structure and the parallel-series structure estimated by the RBD model were larger in the four battery pack configurations. It can also be determined that the more complex the pack structure, the greater the degree of dependency, and the larger the error value of the battery reliability estimation. The RBD model is a simple calculation method that considers the structural relationship between the individual cells without considering the dependency among cells. However, the reliability results obtained from the RBD model were quite different from the actual reliability, which could not accurately evaluate the real reliability of the battery packs or affect the reliability design of the follow-up system (including the lithium-ion battery pack) and the health management of the battery packs. Therefore, we had to quantify the degree of dependency and establish a reliability model that considered the dependency among cells based on the quantified results.

## 3. Method of Modeling the Reliability While Considering the Dependency

Based on the Copula function, the degree of dependency among cells was quantitatively described in order to establish a model that accurately depicted the reliability of the battery pack. Based on this, dependency models of the lithium-ion battery packs with various structures were constructed, and the optimal expression of the model was studied and determined.

### 3.1. A Copula-Based Approach to Quantifying Dependency

The concept of a “Copula” was first proposed by Abe Sklar [22], which is a mathematical tool that joins a marginal distribution with a joint distribution. The Copula function is a connection function of standard uniform random variables:

(5) $$C\left(u_{1},\cdots ,u_{n}\right)=\mathrm{Pr}\left\{U_{1}\leq u_{1},,\cdots ,U_{n}\leq u_{n}\right\}$$

where $U_{i}∼U\left(0,1\right),i=1,2,\cdots n,$ is a standard uniform distribution. The Copula function obeys the Sklar theorem. For the marginal distribution function $F_{1},F_{2},\cdots ,F_{n}$, there is an n-dimensional Copula function C that is satisfied [22]:

(6) $$F\left(x_{1},x_{2},\cdots ,x_{n}\right)=C\left(F_{1}\left(x_{1}\right),F_{2}\left(x_{2}\right),\cdots F_{n}\left(x_{n}\right)\right)$$

If $F_{1},F_{2},\cdots ,F_{n}$ are continuous functions, then there exists a unique C. If $F_{1},F_{2},\cdots ,F_{n}$ represents the marginal distribution function and C is the corresponding Copula function, then the function F defined by Equation (6) is the joint distribution function of $F_{1},F_{2},\cdots ,F_{n}$. The joint probability density function can be obtained by taking the derivative of Equation (7):

(7) $$f\left(x_{1},x_{2},\cdots ,x_{n}\right)=c\left(F_{1}\left(x_{1}\right),F_{2}\left(x_{2}\right),\cdots F_{n}\left(x_{n}\right)\right)\cdot \prod_{i=n}^{n}f_{i}\left(x_{i}\right)$$

where $c\left(u_{1},u_{2},\cdots u_{n}\right)=\frac{\partial^{n}C\left(u_{1},u_{2},\cdots u_{n}\right)}{\partial u_{1}\partial u_{2}\cdots \partial u_{n}}$, $u_{i}=F_{i}\left(x_{i}\right)$, and $f_{i}\left(x_{i}\right)$ is the marginal probability density function of *x_i_*. It can be seen from the Sklar theorem that if the specific form of the Copula function is known, the multidimensional joint distribution function can be constructed flexibly. Several works have studied the forms of the Copula function, among which the Archimedes Copula is one of the most important forms. The Archimedes Copula has advantages of a simple form, symmetry, and combination; therefore, it has been widely used [11]. The Archimedes Copula contains a variety of function types according to the generator elements, which are used to describe different dependent structures, such as the Clayton Copula, Frank Copula, and Gumbel-Hougaard Copula, among others. The Clayton Copula is simple in construction and it can describe a wide range of correlations. Therefore, the Clayton Copula was selected to quantify the dependency among cells of the lithium-ion battery packs.

Assuming that a battery pack is composed of n cells, and that the life of each cell is a random variable, there are *n* marginal cumulative distribution functions (CDF): $F_{i},i=1,2,\ldots ,n$, and a joint CDF: $F_{0}\left(t_{1},t_{2},\ldots ,t_{n}\right)$. The marginal CDF of the random variable is connected to its associated CDF by the Copula function, as shown in Equation (8) [23]:

(8) $$F_{0}\left(t_{1},t_{2},\ldots ,t_{n}\right)=C\left(F_{1}\left(t_{1}\right),F_{2}\left(t_{2}\right),\ldots ,F_{n}\left(t_{n}\right)\right)$$

If $F_{i}$ is continuous, Copula C is uniquely determined. Letting $u_{i}=F_{i}\left(t_{i}\right)$, then the Copula function can be expressed as:

(9) $$C\left(u_{1},u_{2},\ldots ,u_{n}\right)=F_{0}\left(F_{1}^{-1}\left(u_{1}\right),F_{2}^{-1}\left(u_{2}\right),\ldots ,F_{n}^{-1}\left(u_{n}\right)\right)$$

where $u_{1},u_{2},\ldots ,u_{n}\in \left(0,1\right)$. According to the Archimedes Copula, the following specific expressions are obtained:

(10) $$C\left(u_{1},u_{2},\ldots ,u_{n}\right)=\varphi^{\left[-1\right]}\left(\varphi \left(u_{1}\right)+\varphi \left(u_{2}\right)+\cdots +\varphi \left(u_{n}\right)\right)$$

where φ is the Archimedes Copula’s Generator. Supposing that $\varphi_{\theta }\left(t\right)=t^{-\theta }-1$ and θ>0, $\varphi_{\theta }\left(t\right)$ is completely monotonous on [0,∞). For n≥2, the Clayton Copula function can be expressed as:

(11) Cθn(u)=(u1−θ+u2−θ+⋯+un−θ−n+1)−1θ

where *θ* denotes the Copula parameters, which can be solved by the Inference Functions for Margins (IFM) [24,25]. Compared with the maximum likelihood estimation, the IFM has more advantages for numerical calculations and the progressive effect.

After estimating the parameter θ, the joint probability cumulative distribution $F_{0}$ (hereinafter referred to as the dependent term) of the degradation data of the battery packs under dependency is obtained. The dependency degree between n cells is included in parameter $F_{0}$, and the reliability of the battery packs can be obtained from $R=1-F_{0}$.

**Figure 4:**
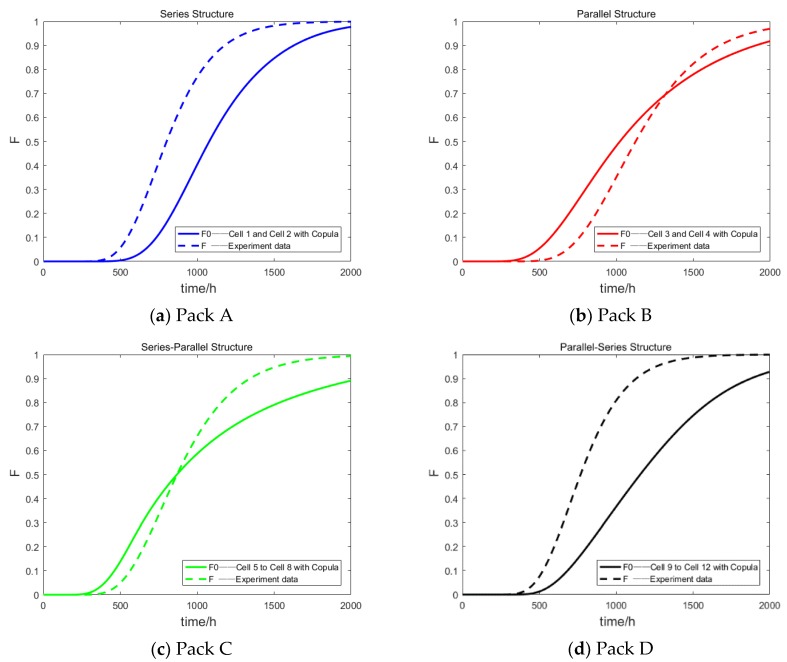
The dependency among individual batteries of four battery packs: (**a**) Pack A, **(b**) Pack B, (**c**) Pack C, (**d**) Pack D.

According to the battery degradation data obtained in Section 2.1, the joint probability cumulative distribution of the two cell degradation data in Pack A and Pack B was regarded as a two-dimensional variable. The degrees of dependency for Pack A and Pack B were calculated using the Copula function. At the same time, the actual joint probability cumulative distributions of Pack A and Pack B were calculated using the data for the whole battery pack degradation. The results are shown in Figure 4a,b. Similarly, the joint probability cumulative distributions of the data for the four cell degradations in Pack C and Pack D were regarded as four-dimensional variables. The dependencies among Pack C and among Pack D were obtained. The results were compared with the actual joint probability cumulative distribution of the battery packs. The results are shown in Figure 4c,d.

### 3.2. Reliability Models of the Lithium-ion Battery Considering the Dependency

As described in Section 3.1, the degradation data of the cell was connected with Copula function to measure the degree of dependency in the overall degradation process of the battery, as well as compared with the actual joint probability cumulative distribution obtained from the battery as a whole. The joint probability cumulative distribution obtained by concatenating the unit cells while simultaneously considering the dependent relationship did not accurately represent the actual degradation of the entire battery pack. Schmalstieg and Wang et al. demonstrated that the trend of capacity degradation of lithium-ion batteries could be described by a linear model [2,26]; therefore, this study considered establishing a linear model to depict the reliability level of the battery pack.

First, as described in Section 2.2, the RBD model was used to obtain the joint probability cumulative distribution of the four battery pack configurations. According to a general linear model structure [27,28], a linear model (Model 1) was established and only considered the internal structural relationship of the battery packs $F_{a}$ (hereinafter referred to as the structural term). The expression for this relationship is as follows:

$$F=aF_{a}+c$$

where F is the joint cumulative probability distribution function that was constructed, and simultaneously considers the internal structural relationship of the battery pack. $F_{a}$ is the structural term obtained by the RBD model, a denotes a weight coefficient term, and c denotes a constant term.

Next, based on the dependent term obtained by the Copula function in Section 3.1, a linear model (Model 2) that considered only the internal dependency among cells was established. The expression for this linear model is:

$$F=aF_{0}+c$$

where F is the joint cumulative probability distribution function that was constructed considering the dependency among cells, $F_{0}$ is the dependent term obtained by the Copula function, a denotes a weight coefficient term, and c denotes a constant term.

**Figure 5:**
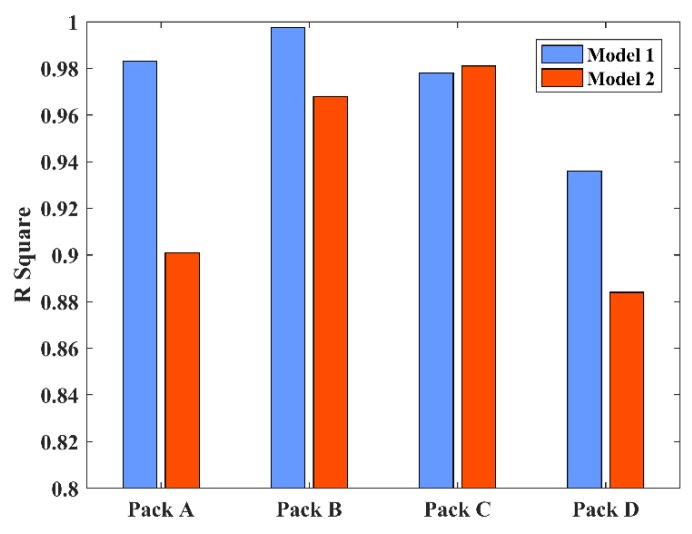
The regression coefficients of Model 1 and Model 2.

In order to verify the ability of the model to describe the degradation law of lithium-ion battery packs under the influence of dependency, the regression analyses of Model 1 and Model 2 were carried out based on the actual test data. The a and c parameters were estimated by the least squares method to obtain the regression model. The significance of the regression equation was tested by the F test. There was a hypothesis H_0_: the model could be expressed in linear form. The alternative hypothesis was H_1_: the model could not be expressed in linear form. When the significance of F was lower than the significance level α=0.01 (*p*-value <0.01), the hypothesis H_0_ was rejected, and the alternative hypothesis, H_1,_ was supported. In addition, *R*^2^ (the regression coefficient) for the goodness of fit was used to test the fitting degree of the actual test data [29]. The larger *R*^2^ value, the greater the proportion of the total variance of the dependent variable was "explained" by the linear regression model. Table 4 provides the parameters and significance of the linear regression models for the four battery packs under Model 1 and Model 2. In order to make the comparison clearer, the regression coefficients of the two models are compared in Figure 5.

The fitting results of Model 1 for Pack A, Pack B, and Pack D was better than that of Model 2, while the fitting result for Pack C was worse than that of Model 2. In Model 2, the regression results obtained by the Copula function were slightly worse, but there were clear advantages in quantifying the degree of dependency of the degradation data of the cells. Therefore, considering the advantages of the Copula function at the level of quantitative dependency and in combination with the RBD model, a new Model 3 was constructed that not only included dependency, but also considered the structure of the battery packs.

In Model 3, the dependent term and the structural term were simultaneously considered as variables affecting the battery pack dependency model; thus, Model 3 had the following three forms of expression:

$$\mathrm{Model}\;\text{3-1}:\;F=F_{a}+aF_{0}+c\;\mathrm{Model}\;\text{3-2}:\;F=a(F_{a}+F_{0})+c\;\mathrm{Model}\;\text{3-3}:\;F=aF_{a}+bF_{0}+c$$

Model 3-1 only considered the weight of the joint cumulative probability distribution function obtained by adjusting the Copula function. Model 3-2 considered the notion that the structural term $F_{a}$ and the dependent term $F_{0}$ had the same weight coefficients. Model 3-3 gave different weights to the structural and dependent terms. The three models listed above only used the Copula function to find the dependent term. In order to understand whether the dependent term $F_{0}$ and the structural term $F_{a}$ had an interactive relationship, the Copula function was used to link the dependent and structural terms to form the following two model structures:

$$\mathrm{Model}\;\text{3-4}:\;F=aC(F_{a},F_{0})+c\;\mathrm{Model}\;\text{3-5}:\;F=aF_{a}+bF_{0}+C(F_{a},F_{0})+c$$

Model 3-4 was a linear model that used the Copula function twice to construct the dependent term as a variable, whereas Model 3-5 regarded the combination of the structural term, the dependent term, and the combination term as multiple variables of the linear model.

The regression analyses of the multiple dependent models of Model 3 were carried out according to the actual experimental data. The dependent models were fitted and their parameters were determined. Additionally, Model 1 and Model 2 were also compared to the modeling effects of the five expression structures of Model 1, Model 2, and Model 3. The best regression model was selected as the lithium-ion battery pack model that considers dependency among cells.

### 3.3. Model Comparison and Discussion

The five expression structures of Model 3 were analyzed with regression analysis. The model parameters of Pack A, Pack B, Pack C, and Pack D were estimated, and the model was tested for significance and goodness of fit, as shown in Table 5.

**Figure 6:**
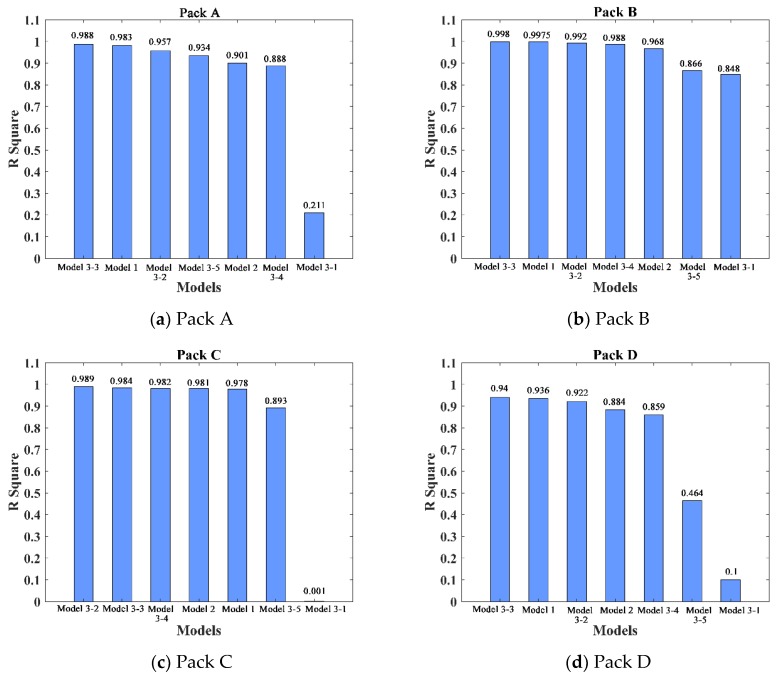
The results of the fitting effects of each model for the four battery packs: (**a**) Pack A, (**b**) Pack B, (**c**) Pack C, (**d**) Pack D.

In order to visually illustrate the ability of each model to fit the actual battery pack degradation data, the regression results of the seven models were compared according to the configurations of the battery packs. The comparison results are shown in Figure 6.

By comparing the graphs in Figure 6, it can be seen that the multivariate linear models (Model 3-2 and Model 3-3) that considered both the dependent and structural terms had a better fitting effect than Model 1 and Model 2. Models 3-2 and 3-3 also had high regression coefficients (*R*^2^>0.9) and low *p*-values (<0.0001) for the four battery pack configurations. The regression effect was remarkable, which suggests that the model structure containing the dependent term and the battery pack structural term was more suitable for expressing the dependent relationship among cells. The fitting result of Model 3-3 was better than that of Model 3-2 in Pack A, Pack B, and Pack D, and second only to Model 3-2 in Pack C. From these results, it is clear that the model with different weight coefficients for the dependent and structural terms was more reasonable. Therefore, the final reliability dependency model of the lithium-ion battery pack was obtained by choosing the expression of Model 3-3, i.e.,:

$$R=aR_{a}+b\left(1-F_{0}\right)+c$$

This model described the reliability level of the entire battery pack by combining the reliability of a single battery with the dependency among cells into the model, which more accurately indicated the reliability of the entire pack. The model construction idea presented in this work provided a new solution for the reliability evaluation of a system with a dependent relationship. The state of the entire system can be represented in combination with the state of each component, especially for a system in which the state of each component is easy to obtain and the overall state of the system is difficult to acquire.

## 4. Application in Battery Group Design

The reliability model that considers dependency among cells presented in Section 3 can provide guidance for scheme optimization, adjustment, and the reliability evaluation of battery packs in the reliability design stage of electric drive products.

**Figure 7:**
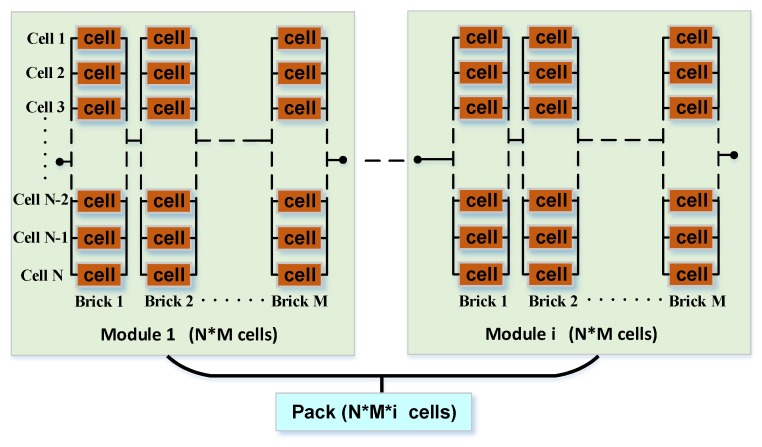
The structure of the battery system of the Tesla Model S.

As an example, the battery system of the Tesla Model S consists of a large number of 18,650 lithium-ion batteries in series and in parallel. The typical grouping structure is shown in Figure 7. A number of N cells are connected in parallel to form a Brick, a number of M Bricks are connected in series to form a Module, and a number of i Modules are connected in series to form a Pack.

In the design phase, our goal was to select a group of cells with the least dependency and highest reliability to form a pack structure, based on the premise of meeting the overall output voltage and energy requirements. The preferred method for the battery pack structure is to obtain the capacity degradation data of a set of Bricks and calculate the reliability of each Brick according to the reliability dependency model in Section 3.3. Due to the existence of the equalizer, and assuming that there is no dependency between Bricks, the reliability of the overall Pack can be calculated by the reliability model. The calculation equations are as follows:

(12) $$R_{Brick}=a\left(1-\prod_{1}^{N}\left(1-R_{j}\right)\right)+b(1-F_{0})+c$$

(13) $$R_{Pack}=\prod_{1}^{i}\prod_{1}^{M}R_{Brick}$$

The numbers of N, M, and i are changed to achieve the battery group structure with the highest reliability that accounts for dependency among cells. At the same time, in the usage phase, the reliability level of the battery pack can be accurately evaluated based on the accumulated battery degradation data. Due to various restrictions on the operating environment as well as cost, this study only tested the battery packs of the basic group structure. For the complex structure (Figure 7), we propose a possible solution. The testing and verification of similar complex groups will be the focus of future research.

## 5. Conclusions

This work researched a reliability model for lithium-ion battery packs that considers dependency among cells. Based on existing research [2], the reliability of a lithium-ion battery pack was analyzed by obtaining test data. The influence of dependency on the reliability of the battery pack was verified and quantified. The reliability model of a battery pack that considers dependency was constructed. The proposed model provided a viable optimization idea for battery group design. The main conclusions were as follows:

(1) For the four battery pack configurations, there was a significant difference between the reliability obtained using the RBD model of the cells and the reliability obtained using the actual battery pack test data. The dependency among cells for the overall degradation process accelerated the degradation rate of the entire battery pack and affected the reliability of the entire battery pack.

(2) Linear models, Model 1, Model 2, and Model 3 (including the five sub-models), were established. Three types of models were compared in the four groups of structures according to the fitting effect. The expression of Model 3-3 was more capable of describing the dependent relationship. Therefore, based on Model 3-3, the final battery pack reliability model was established.

(3) Based on the model described in this paper, a feasible optimization idea for battery group design of large electric drive products is provided. However, only four battery pack configurations were tested in this study. Additional testing and verification of complex battery packs will be carried out in future work.

## Figures and Tables

**Table 1:** The failure threshold for the cell and the four battery packs.

Cell and Packs	Rated Capacity (Ah)	Failure Threshold Value *w* (Ah)
Cell	2.15	0.43
Pack A	2.15	0.43
Pack B	4.30	0.86
Pack C	4.30	0.86
Pack D	4.30	0.86

**Table 2:** Reliability functions of the four battery packs.

Pack	*R*
Pack A	$R=R_{1}\cdot R_{2}$
Pack B	$R=1-(1-R_{3})\cdot (1-R_{4})$
Pack C	$R=1-(1-R_{5}\cdot R_{6})\cdot (1-R_{7}\cdot R_{8})$
Pack D	$R=(1-(1-R_{9})\cdot (1-R_{10}))\cdot (1-(1-R_{11})\cdot (1-R_{12}))$

**Table 3:** The RMSE values of the reliability estimated by the test data and the reliability estimated by the RBD model.

Pack	Pack A	Pack B	Pack C	Pack D
RMSE	0.0674	0.0648	0.0821	0.1377

**Table 4:** Parameters and significance of the linear regression models for the four battery packs under Model 1 and Model 2.

Models	Pack	a	c	*R* ^2^	p-Value
Model 1	Pack A	0.953	0.061	0.983	<0.01
	Pack B	1.119	0.004	0.9975	<0.01
	Pack C	0.984	−0.047	0.978	<0.01
	Pack D	0.927	0.121	0.936	<0.01
Model 2	Pack A	1.02	0.139	0.901	<0.001
	Pack B	1.101	−0.074	0.968	<0.001
	Pack C	1.301	−0.033	0.981	<0.001
	Pack D	1.13	0.142	0.884	<0.001

**Table 5:** Parameters and significance of the linear regression model of the four battery packs under Model 3.

Model 3	Pack	*a*	*b*	*c*	*R* ^2^	p-Value
Model 3-1	Pack A	−0.068	/	0.065	0.211	<0.01
	Pack B	0.121	/	−0.007	0.848	<0.01
	Pack C	0.007	/	−0.06	0.001	<0.01
	Pack D	−0.1	/	0.124	0.1	<0.01
Model 3-2	Pack A	0.5	/	0.092	0.957	<0.01
	Pack B	0.56	/	−0.04	0.992	<0.01
	Pack C	0.566	/	−0.47	0.989	<0.01
	Pack D	0.514	/	0.126	0.922	<0.01
Model 3-3	Pack A	1.229	−0.317	0.048	0.988	<0.01
	Pack B	1.003	0.117	−0.006	0.998	<0.01
	Pack C	0.401	0.732	−0.046	0.984	<0.01
	Pack D	1.181	−0.328	0.121	0.94	<0.01
Model 3-4	Pack A	1.017	/	0.148	0.888	<0.01
	Pack B	1.261	/	0.014	0.988	<0.01
	Pack C	1.291	/	−0.011	0.982	<0.01
	Pack D	1.102	/	0.169	0.859	<0.01
Model 3-5	Pack A	0.197	0.001	−0.011	0.934	<0.01
	Pack B	−0.018	0.26	−0.011	0.866	<0.01
	Pack C	0.507	−0.363	−0.03	0.893	<0.01
	Pack D	1.187	−1.338	0.139	0.464	<0.01
